# A Hospitality Improvement Intervention in Residential Care Does Not Warrant Staff Job Satisfaction or Turnover Intention: A Cross-Sectional Survey Study Investigating the Hostmanship Program

**DOI:** 10.7759/cureus.23601

**Published:** 2022-03-29

**Authors:** Hanan Daghash, Filip Haegdorens, Katrin Gillis, Stijn Slootmans, Koen De Smedt, Peter Van Bogaert

**Affiliations:** 1 Nursing, Al-Ghad International Health Sciences Colleges, Tabuk, SAU; 2 Nursing and Midwifery, University of Antwerp, Antwerpen, BEL; 3 Nursing, Odisee University College, Sint-Niklaas, BEL

**Keywords:** quality improvement, residential care settings, nurse turnover, work engagement, job satisfaction, burnout

## Abstract

Background

Accountability pressure is rising in healthcare, and this demonstrates that the quality of care provided within a residential care setting is of utmost importance. Hostmanship is a quality improvement program focusing on person-centered care in residential care settings.

Objectives

This study aimed to explore the influencing factors for job satisfaction and intention to leave among healthcare workers and the difference in job satisfaction and intention to leave the employer between residential care centers with and without Hostmanship.

Methods

A quantitative, cross-sectional study was conducted in sixteen Flemish residential care settings in Belgium. A total of 293 participants completed the questionnaire, divided into two groups: the group with Hostmanship (n = 139), at least one year into a change process implementing Hostmanship, and the group without the Hostmanship program (n = 154). Hierarchical logistic regression analysis estimated effects between demographic characteristics (block one), facility management, staffing and Hostmanship (block two), work characteristics (block three), and work engagement or burnout dimensions (block four) as explanatory variables of job satisfaction and turnover intention as outcome variables.

Results

This study confirmed the positive impact of social capital and decision latitude on staff member job satisfaction, as shown in previous findings. Age and workload were associated with turnover intentions. A hierarchical logistic regression model explained 68.7 % of the variance in workers' job satisfaction, and a hierarchical logistic regression explained 49.2% of the variance in their intent to leave. Also, no effects were found for Hostmanship on staff job satisfaction and intention to leave.

Conclusions

This study shows how a quality improvement project such as Hostmanship could produce counterintuitive results for organizations in elderly residential centers. However, results inconsistent with literature were found. It is unclear whether Hostmanship warrants job satisfaction or retaining personnel. Future research must take into consideration success factors when implementing new quality initiatives. A general framework for successful implementation in the healthcare sector should be provided.

## Introduction

In today's highly competitive health care environment, each member of the health care organization must be accountable for the quality and cost of health care. After the publication of the Institute of Medicine's (IOM) reports on medical errors in 1999 (IOM, 1999) and their later recommendations for health professionals' education and practices, concerns about quality gained national and international attention [1]. Additionally, concerns about cost continue unabated. Both quality and cost containment are found in the concept of total quality management (TQM), which has evolved into a model of continuous quality improvement designed to improve the system and process performance [2]. TQM, also referred to as quality improvement and performance improvement, which began in the manufacturing industry with William Edwards Deming and Joseph Juran (1986) [3], has further developed with new concepts and empirical insights, such as in human factors and ergonomics, lean thinking as well as high-reliability organizations in healthcare [4].

Approaches and methods for improving outcomes are increasingly being accepted as critical strategies to minimize health inequalities and maximize healthcare programs [5]. Some strategies to help make healthcare institutions more productive have been shown to improve efficiency and quality. However, it requires several initiatives more complex than a single initiative to enhance quality in teams or quality work in one department [6]. These programs create conditions that hinder smaller quality improvement projects [7]. Although the strong growth in the use of various quality improvement initiatives, there is limited knowledge about the efficacy of these programs. Some studies show an improvement in patient outcomes, while others show modest or even no effect [8]. These diffuse results may possibly be explained by differences in the context in which these initiatives are implemented [8]. These differences may be situated in the need to use an organization-specific implementation program and structure for success [9]. In addition, other researchers reported motivation and commitment of healthcare workers, support with data collection, leadership, and changing behavior as explanatory factors with a major impact [9].

Besides, there are increasing concerns about the amount of resources being devoted to these interventions without being certain they are improving the quality of care. Sometimes, it is useful to generate knowledge from quantitative research on innovation, such as with a cross-sectional or network analysis [10]. This information given by quantitative research is believed to be valuable to so-called practitioners, providing knowledge that is useful beyond the science world. A cross-sectional study is a type of quantitative research that is designed to examine the impact of a program but does not reflect substantially on the causes for the result. This type of study seeks to help program directors or policymakers determine whether to discard, substitute, modify, maintain, or repeat the program or regulation.

Residential programs face increasing accountability and performance measurements tasks, as the probability of requiring residential care settings rises exponentially with age. Hostmanship is an example of a program aiming to improve the practice environment in residential care settings. In particular, Hostmanship is a concept that originated in Sweden in 2003 as an organizational culture with good hospitality as a shared goal [11]. The concept is described by the Hostmanship group as "the art of making people feel welcome". The concept must provide a distinctive experience and create added value that translates into loyal 'customers' and proud employees [11]. The main characteristic of the concept is that everyone is considered a guest. Because of this specific approach, the organization's attractiveness for employees could increase [11,12]. The agreement is that the implementation aims to increase the satisfaction of employees and clients and, by extension, maintain or increase the market share and attract and retain good contributors [11]. Furthermore, Hostmanship aims to share a vision to pursue responsibility specifically geared to the residents' needs. It leaves the care provider free to decide how the needs of the residents can be met [11,12]. Hostmanship is, therefore, a form of person-centered care. In nursing homes, the concept of person-centered care is defined by Vassbø et al. (2019) [13] as "staff were able to meet individual resident's needs and expressed preferences in close family-like relationships, understanding the residents' rhythms and preferences as the basis of the daily work plans and being able to do 'the little extra' for residents". Also, working in a person-centered way meant meeting shared goals by working towards a collective practice in collaborative teams."

Residential care settings are considered a last option for individuals who can no longer live at home. These individuals face a high level of care need, which can create a complex and unpredictable environment. For this reason, it is highly important that employees in this environment are supported and retained over time to utilize their knowledge for the benefit of other employees as well as the client. This can be done by investing in person-centered care and organizational culture [14]. There is international evidence suggesting that person-centered care has a favorable impact on staff involvement and job satisfaction [15]. In addition, person-centered care has a positive influence on resident satisfaction [16]. For example, the implementation of person-centered care for elderly people with dementia has a positive influence on their quality of life, resulting in less agitation, neuropsychiatric symptoms, and depression [17]. Momentarily, there is no scientific research on the impact of Hostmanship itself. The aim of the study is twofold: what are the influencing factors for job satisfaction and intention to leave among healthcare workers in residential care settings?, and is there any difference in job satisfaction and intention to leave the employer between residential care centers with and without Hostmanship?

## Materials and methods

Design

A cross-sectional study design was used. Participants were invited by a coordinator/contact person at each residential aged center to voluntarily complete questionnaires. An online self-report questionnaire via Qualtrics XM (Qualtrics, Seattle, Washington) was used to collect the data between December 13, 2018, and March 15, 2019, in Flemish residential care settings in Belgium. In one residential care setting, it was technologically impossible to access the digital questionnaire; the questionnaire was administered on paper.

Participants and study setting

Data collection took place in 16 Flemish residential care centers, all incorporated in a privately organized group approved and funded by the Flemish Agency for Care and Health. A convenience sample was used to involve all direct caregivers such as nurses and licensed nurse practitioners. Eight residential care centers included used Hostmanship, which was introduced more than one year ago as an organizational culture and practice change (residential care centers with Hostmanship), and eight residential care centers were included where Hostmanship is unknown (residential care centers without Hostmanship). The implementation strategy of Hostmanship was twofold: firstly, leadership and management learned about the concept of Hostmanship. Secondly, nursing staff were introduced to the principles of Hostmanship, supported by interactive workshops. The implementation was intuitive and not guided by a scientific approach to investigating staff readiness to change practice and culture. Also, the extent to which their practices need to be improved or changed was not explored.

Research instruments

The questionnaire was composed of validated, tested, and well-used instruments in a previous study [18]. In order to provide a survey with a feasible number of items, researchers made a content-driven selection of some subscales of the Revised Nursing Work Index (nurse management at the team level and facility management and support) as studied in a previous study measuring nurse practice environment with four subscales: nurse-physician relations (three items), nurse management at the team level (three items), facility management and support (three items), and staffing adequacy (two items). Furthermore, nurse work characteristics were measured with three subscales related to the concept of staff empowerment: encompassed workload (six items), decision latitude (seven items), and social capital (six items). In both measures, the staff were asked to rate their agreement with various statements in their current positions on a four-point Likert-type scale (i.e., strongly disagree, disagree, agree, strongly agree). Burnout as the negative and engagement as the positive pole of a continuum were measured with Maslach Burnout Inventory Human Service Survey (MBI-HSS) with three subscales: emotional exhaustion (seven items), depersonalization (five items), and personal accomplishment (eight items), and the Utrecht Work Engagement Scale (UWES) with three subscales: vigor (three items), dedication (three items) and absorption (three items), respectively. Respondents rated their experience of various job-related feelings on a seven-point and five-point Likert-type scale, respectively, ranging from 'never' to 'every day'. Nursing-reported job outcomes were measured using two items: satisfaction with the current job ((very) dissatisfied, (very) satisfied), and intention to leave the profession (yes, no). All variables, with the exception of workload, emotional exhaustion, and depersonalization, were coded for analysis, with higher scores indicating stronger agreement or more favorable ratings.

Data analysis

Demographic characteristics were collected, such as age, gender, work schedule, and function. Percentages were reported for categorical variables and the mean and the standard deviation for continuous variables. The Mann-Whitney U test was performed in order to study differences between without Hostmanship and with Hostmanship groups. Based on previous studies, nurse practice environment dimensions, nurse work characteristics, engagement, and burnout dimensions were treated as explanatory variables [19-21]. Hierarchical logistic regression analysis estimated the strength of the associations with demographic characteristics such as age, gender, work schedule and function (block one), facility management, staffing and Hostmanship (block two), work characteristics (decision latitude, social capital, and workload) (block three) and work engagement or burnout dimensions (block four) as explanatory variables of job satisfaction and intention to leave as outcome variables. For the logistic regression analysis, job satisfaction was dichotomized into categories (1=(very) satisfied, 0=(very) dissatisfied), the intention to leave was also dichotomized into categories (1=yes, 0=no). A statistical significance level of p<.05 was set, and the Statistical Package for the Social Sciences version 25.0 (IBM Inc., Armonk, New York) software was used for all the analyses.

## Results

A total of 293 participants were reached (response rate of 57%), with 139 participants in the nursing home with the Hostmanship group and 154 participants in the nursing home without the Hostmanship group (Table 1). Most of the study participants were female, and the average age was about 35 years old. More than half of the participants worked as licensed nurse practitioners, and almost one out of four were registered nurses. The percentage of respondents that were satisfied with their current job in the without Hostmanship group and with Hostmanship group was 84.4% and 84.9%, respectively. The percentage of respondents who intended to leave the profession was 15.6% in the group without Hostmanship and 23.7% in the group with Hostmanship.

**Table 1:** Demographics and study variables

	Without Hostmanship (n=154)		With Hostmanship (n=139)	
	N	%	N	%
Gender				
Male	22	14.3	18	12.9
Female	132	85.7	121	87.1
Function				
Registered nurses	37	24.1	31	22.3
Healthcare staff	33	21.4	31	22.3
Licensed practical nurses	84	54.5	77	55.4
(Very) satisfied with the current job (Yes)	130	84.4	118	84.9
Intention to leave the profession (Yes)	24	15.6	33	23.7

Mann-Whitney U test was performed in order to study differences between without Hostmanship and with Hostmanship groups. There were five statistically significant differences found it. A summary of the mean scores between the two groups is presented in Table 2. For facility management support, we observed participants in the group without Hostmanship scored a significantly higher mean value compared with the group with Hostmanship mean value (3.00 versus 2.85). In addition, staffing perception was rated as inadequate with significantly lower mean values in the group with Hostmanship compared with the group without Hostmanship (2.03 versus 2.25). The mean value of workload for the group without Hostmanship participants was significantly lower than the mean value of the group with Hostmanship (2.86 versus 3.03). Staff rated feelings of emotional exhaustion and depersonalization were low and significantly lower in the group without Hostmanship, compared with the group with Hostmanship, with mean values of 1.35 versus 1.84, and 0.58 versus 1.01, respectively. Vigor, dedication, and absorption were rated high with higher mean values in the group without Hostmanship, compared to the group with Hostmanship mean values of 4.78 versus 4.57, 4.95 versus 4.79, and 4.89 versus 4.72, respectively.

**Table 2:** Mann-Whitney U test comparing between residential care centers without Hostmanship and with Hostmanship *p-value< .05; **p-value< .01

	Without Hostmanship (154)		With Hostmanship (139)	
	Mean	SD	Mean	SD
Age	35.68	10.45	35.75	12.02
Work schedule	78.64	20.55	75.60	20.70
Facility management	3.00	.44	2.85	.49*
Staffing	2.25	.84	2.03	.69*
Decision latitude	2.98	.34	2.93	.38
Social capital	2.89	.63	2.95	.50
Workload	2.86	.56	3.03	.49**
Emotional exhaustion	1.35	1.54	1.84	1.72*
Depersonalization	0.58	.84	1.01	1.29**
Personal accomplishment	4.96	1.20	5.17	.88
Vigor	4.78	1.22	4.57	1.33
Dedication	4.95	1.26	4.79	1.21
Absorption	4.89	1.19	4.72	1.30

Hierarchical logistic regression analyses explained variances were 57.6% for the satisfaction with the current job (Table 3) in the burnout models. Furthermore, hierarchical logistic regression analyses explained variances were 68.7% for the satisfaction with the current job (Table 4) in engagement models. In engagement models, work characteristics (block three) explained 49.4 % of the variance in the satisfaction with the current job, indicating a rise of one point in the decision latitude, and social capital score increases the odds of job satisfaction with 18.5 and 4.62, respectively. Significant negative associations were estimated (block four) between job satisfaction and emotional exhaustion in the burnout model with odds of almost 65% (0.36) (Table 3) and positive associations between job satisfaction and dedication in the engagement model with odds of 5.47 (Table 4).

**Table 3:** Hierarchical logistic regression analysis Hierarchical logistic regression analysis estimated the strength of the associations with demographic characteristics such as age, gender, work schedule and function (block one), facility management and Hostmanship (block two), work characteristics (block three), and burnout dimensions (block four) as an explanatory variable of job satisfaction as the dependent variable. *p-value< .05; **p-value< .01; females as indicators; Hostmanship as an indicator; licensed practical nurses as indicators; gender (1) - male; function (1) - registered nurses, function (2) - healthcare staff; Hostmanship (1) - not implemented; B - B coefficient; SE - standard error

Job satisfaction: (very) satisfied (1) versus (very) dissatisfied (0)	B	SE	OR	95% C.I.		adjR²
				Lower	Upper	
Age	.037	.035	1.038	.968	1.112	
Gender (1)	.391	.914	1.478	.247	8.861	
Work schedule	.031	.019	1.032	.994	1.071	
Function						
Function(1)	-2.295	1.055	.101*	.013	.797	
Function(2)	-.985	1.163	.373	.038	3.649	.146
Facility management	1.370	.867	3.934	.720	21.502	
Staffing	1.000	.742	2.719	.635	11.637	
Hostmanship (1)	.150	.858	1.161	.216	6.236	.351
Decision latitude	2.444	1.365	11.514	.793	167.246	
Social capital	.872	.691	2.391	.617	9.265	
Workload	1.625	1.100	5.079	.588	43.897	.490
Emotional exhaustion	-1.017	.363	.362**	.178	.736	
Depersonalization	.454	.346	1.574	.798	3.103	
Personal accomplishment	.429	.350	1.536	.774	3.049	.576

**Table 4:** Hierarchical logistic regression analysis Hierarchical logistic regression analysis estimated the strength of the associations with demographic characteristics such as age, gender, work schedule and function (block one), facility management and Hostmanship (block two), work characteristics (block three), and work engagement dimensions (block four) as an explanatory variable of job satisfaction as the dependent variable *p-value< .05; **p-value< .01; females as indicators; Hostmanship as an indicator; licensed practical nurses as indicators; gender (1) - male; function (1) - registered nurses, function (2) - healthcare staff; Hostmanship (1) - not implemented; B - B coefficient; SE - standard error

Job satisfaction: (very) satisfied (1) versus (very) dissatisfied (0)	B	SE	OR	95% C.I.		adjR²
				Lower	Upper	
Age	.037	.040	1.037	.960	1.122	
Gender (1)	.185	1.133	1.204	.131	11.094	
Work schedule	.022	.021	1.022	.980	1.065	
Function						
Function (1)	-3.419	1.250	.033**	.003	.380	
Function (2)	-.488	1.356	.614	.043	8.762	.115
Facility management	.971	.847	2.642	.502	13.897	
Staffing	1.188	.864	3.279	.603	17.818	
Hostmanship (1)	1.022	.946	2.778	.435	17.730	.289
Decision latitude	2.917	1.257	18.485**	1.575	216.969	
Social capital	1.530	.775	4.616*	1.012	21.065	
Workload	.730	.995	2.075	.295	14.575	.494
Vigor	.502	.442	1.652	.694	3.933	
Dedication	1.699	.662	5.468	1.494	20.011	
Absorption	-.864	.465	.421	.169	1.049	.687

Hierarchical logistic regression analyses explained variances were 40.8% for intention to leave the profession (Table 5) in the burnout models. Furthermore, hierarchical logistic regression analyses explained variances were 49,2% for intention to leave the profession (Table 6) in engagement models. In engagement models, work characteristics (block three) explained 49.4 % of the variance in the satisfaction with the current job, indicating a rise of one point in the decision latitude, and social capital score increases the odds of job satisfaction with 18.5 and 4.62, respectively. Moreover, in both models, workload (block three) was significantly associated with intention to leave with odds of 4.8 and 5.1 - an increase of one point in workload increases the likelihood of employee departure (Table 5, 6). Participants’ age was negatively associated with the intention to leave the profession in both models, with 23.2% explained variance (block one) in the engagement model compared with 20.3% in the burnout model (Table 5, 6). No effects were found for Hostmanship on staff job satisfaction and intention to leave in the burnout as well as engagement model.

**Table 5:** Hierarchical logistic regression analysis Hierarchical logistic regression analysis estimated the strength of the associations with demographic characteristics such as age, gender, work schedule and function (block one), facility management and Hostmanship (block two), work characteristics (block three), and burnout dimensions (block four) as an explanatory variable of turnover intention as the dependent variable. *p-value< .05; **p-value< .01; females as indicators; Hostmanship as an indicator; licensed practical nurses as indicators; gender (1) - male; function (1) - registered nurses, function (2) - healthcare staff; Hostmanship (1) - not implemented; B - B coefficient; SE - standard error

Intention to leave the profession: yes (1) versus no (0)	B	SE	OR	95% C.I.		adjR²
				Lower	Upper	
Age	-0.105	0.028	.901**	0.853	0.951	
Gender (1)	-1.021	0.573	0.36	0.117	1.107	
Work schedule	0.007	0.012	1.007	0.985	1.031	
Function						
Function (1)	0.07	0.653	1.073	0.299	3.855	
Function (2)	0.102	0.602	1.108	0.34	3.607	0.203
Facility management	-0.015	0.491	0.985	0.376	2.577	
Staffing	-0.06	0.359	0.942	0.466	1.902	
Hostmanship (1)	-0.631	0.434	0.532	0.227	1.246	0.251
Decision latitude	-0.976	0.718	0.377	0.092	1.54	
Social capital	0.165	0.44	1.179	0.497	2.796	
Workload	1.561	0.672	4.762**	1.276	17.764	0.39
Emotional exhaustion	0.333	0.194	1.395	0.953	2.04	
Depersonalization	-0.228	0.221	0.796	0.517	1.227	
Personal accomplishment	-0.114	0.212	0.893	0.589	1.353	0.408
Intention to leave the profession: yes (1) versus no (0)	B	SE	OR	95% C.I.		adjR²
				Lower	Upper	
Age	-0.105	0.028	.901**	0.853	0.951	
Gender (1)	-1.021	0.573	0.36	0.117	1.107	
Work schedule	0.007	0.012	1.007	0.985	1.031	
Function						
Function (1)	0.07	0.653	1.073	0.299	3.855	
Function (2)	0.102	0.602	1.108	0.34	3.607	0.203
Facility management	-0.015	0.491	0.985	0.376	2.577	
Staffing	-0.06	0.359	0.942	0.466	1.902	
Hostmanship® (1)	-0.631	0.434	0.532	0.227	1.246	0.251
Decision latitude	-0.976	0.718	0.377	0.092	1.54	
Social capital	0.165	0.44	1.179	0.497	2.796	
Workload	1.561	0.672	4.762**	1.276	17.764	0.39
Emotional exhaustion	0.333	0.194	1.395	0.953	2.04	
Depersonalization	-0.228	0.221	0.796	0.517	1.227	
Personal accomplishment	-0.114	0.212	0.893	0.589	1.353	0.408

**Table 6:** Hierarchical logistic regression analysis Hierarchical logistic regression analysis estimated the strength of the associations with demographic characteristics such as age, gender, work schedule and function (block one), facility management and Hostmanship (block two), work characteristics (block three), and work engagement dimensions (block four) as an explanatory variable of turnover intention as the dependent variable. *p-value< .05; **p-value< .01; females as indicators; Hostmanship as an indicator; licensed practical nurses as indicators; gender (1) - male; function (1) - registered nurses, function (2) - healthcare staff; Hostmanship (1) - not implemented; B - B coefficient; SE - standard error

Intention to leave the profession: yes (1) versus no (0)	B	SE	OR	95% C.I.		adjR²
				Lower	Upper	
Age	-0.114	0.032	.892**	0.838	0.95	
Gender (1)	-1.208	0.641	0.299	0.085	1.049	
Work schedule	0.02	0.013	1.021	0.995	1.047	
Function						
Function (1)	-0.526	0.68	0.591	0.156	2.24	
Function (2)	-0.72	0.723	0.487	0.118	2.009	0.232
Facility management	0.363	0.524	1.438	0.515	4.013	
Staffing	-0.446	0.366	0.64	0.313	1.311	
Hostmanship (1)	-0.623	0.464	0.536	0.216	1.331	0.275
Decision latitude	0.001	0.724	1.001	0.242	4.14	
Social capital	0.135	0.464	1.144	0.46	2.843	
Workload	1.629	0.616	5.098**	1.523	17.068	0.398
Vigor	-0.191	0.368	0.826	0.402	1.699	
Dedication	-0.388	0.344	0.678	0.346	1.331	
Absorption	-0.249	0.287	0.78	0.444	1.369	0.492

## Discussion

This study reveals that Hostmanship does have counterintuitive findings on various dimensions of the professional wellbeing of care professionals in residential care settings. In the hierarchical logistic regression analyses, we examined factors related to healthcare workers’ job satisfaction and intention to leave their residential centers in both engagement and burnout models.

As identified in a literature review, the most strongly predicted determining factors of intention to leave were burnout and commitment; additionally, supervisor support was the most supported determinant for retention [22]. In the present study, intention to leave the profession was positively associated with workload and negatively with the age of employees in burnout and engagement models. This finding broadly supports the previous studies, where young nurses were intent to leave the profession [23], and workload was associated with intention to leave [24]. The current result may be explained by the fact that residents’ dependency might have a striking effect on nursing workload. Therefore, it seems that the dependency level of residents in long-term facilities on needs and staff requires more time for nursing activities [25]. To develop a full picture of prediction factors on intention to leave a residential aged center, additional longitudinal studies are needed.

The current study results showed that high rates of social capital and decision latitude among nurses were negatively associated with emotional exhaustion and positively associated with dedication in burnout and engagement models, respectively. This study supports evidence from previous observations [19-21]. In addition, the results of this study indicate the positive impact of high rates of social capital and decision latitude on high rates of job satisfaction. Evidence shows that when nurses are supported to make decisions and that they have the opportunity and capacity to plan, be creative, utilize and develop one’s professional capabilities, job satisfaction increases. This result may be explained by the fact that healthcare workers who feel that their workplace is empowering and who have the necessary support are more likely to feel fulfilled and satisfied. Moreover, the current study confirmed associations between social capital and decision latitude with job satisfaction, regardless of a Hostmanship program. 

Paradoxically, an unanticipated finding was that the prediction factor for intention to leave the profession was different than the prediction factor that contributes to job satisfaction. Prior literature review noted that job dissatisfaction of nurses contributed to their high turnover rate [26, 27]. This finding suggests that the sources of nurses’ job dissatisfaction, its effect, and the related factors affecting nurses’ intention to leave have not yet been conclusively identified. Job satisfaction is a complex phenomenon with many affected components, yielding some inconsistent findings within different cultural settings and values [27]. The current study identifies predictors of job satisfaction and intention to leave, which need further investigation through longitudinal and intervention studies.

What is surprising is that this study does not disclose a significant improvement in job satisfaction and intention to leave between with Hostmanship and without Hostmanship groups. This outcome is contrary to that of Flynn et al. [28], who found that when scientifically substantiated person-oriented interventions are implemented, employees experience increased job satisfaction. The pragmatic reasons for the failure of quality improvement programs are, according to Kovach and Mairani [29], a clear link between organizational context and project failure. White et al. [30] reported that overstretched clinical environments struggle with the right climate and context in using a large-scale quality improvement program in Ireland. Moreover, the current study showed that staffing perception was rated as inadequate in the group with Hostmanship compared with the group without Hostmanship. Hostmanship is a complex social intervention and requires the right climate, conditions, and context to be implemented successfully to produce sustained improvements. Additionally, it may be reasonable to expect no effect on staff-related outcome variables due to a program that focuses on the wellbeing of residents and not so much on the wellbeing of staff. The locally available resources at the point of implementation and adoption can shape the evolving forms of assimilation into routine practice and wider contextual changes of a quality improvement program.

Flynn et al. [28] reported that when person-oriented interventions are successfully implemented, employees experience increased job satisfaction, and there is lower employee turnover, and that willingness of the organization seems critical to the effective implementation of person-centered care. The lesson we draw from this study is to recognize that active ingredients define Hostmanship. Indeed, what are the indicators and domains relevant to Hostmanship? In order to provide a deeper understanding of the concept and if the program is successfully implemented, it could be interesting to develop a validated instrument that measures Hostmanship concepts. In addition, prospective testing approaches are essential to assess and ensure that these active ingredients are correctly applied throughout multiple residential care centers. From an academic point of view, a note of caution is that this program has had a positive economic influence on the hospitality industry and may not have an effect on workplaces in the medical and wellness sectors. However, what works for one organization may not work for another because of subtle differences in these processes and barriers. One overall solution will not fit every organizational challenge, and the ingredients or components of the complex intervention will differ from project to project. Although there is still more work to be done to improve the success rate of the Hostmanship initiative in residential care settings, this discussion has provided a first step in bringing attention to this problem and developing ideas from some interesting perspectives to address it.

The current study provided some relevant insights on quality improvement initiatives such as Hostmanship and predicting factors of staff job satisfaction and turnover intentions. However, some limitations are worthy of attention. First, because of the cross-sectional design, causal effects cannot be demonstrated. The second limitation of the current study was the use of self-report to collect data. Therefore, some errors may have occurred, causing some limitations such as possible social desirability bias. The social desirability bias, where participants will answer the survey in a favorable manner according to how they feel the researcher would want them to respond was also present. Furthermore, this research focuses only on the impact of organizational culture on professional wellbeing. However, the extent to which the new organizational culture has changed was not examined, and it is unclear whether the norms and values of the preceding culture are different from the current one. There may be selection bias and limited generalizability. A longitudinal study where different measurements over time are carried out at the same residential care centers could form a more objective and well-founded judgment here.

## Conclusions

This study offers how using a Hostmanship program could produce counterintuitive results for organizations in elderly residential centers. It is not clear that Hostmanship warrants job satisfaction or retaining personnel. Future research must take into consideration success factors when implementing new quality initiatives. A general framework of the Hosmanship for successful implementation in the healthcare sector should be provided.
